# Supplementary material for: Splice-Junction-Based Mapping of Alternative Isoforms in the Human Proteome
Source: Cell Rep. Author manuscript; Available in PMC 2020 Jan 15. (PMC6961840; doi:10.1016/j.celrep.2019.11.026)
Supplement: 2 [file NIHMS1546469-supplement-2.zip › DF1/PXD006675/AtrialSeptum/AtrialSeptum_2_MYBPC3_RTDSHEDTGILDFSSLLK.pdf]

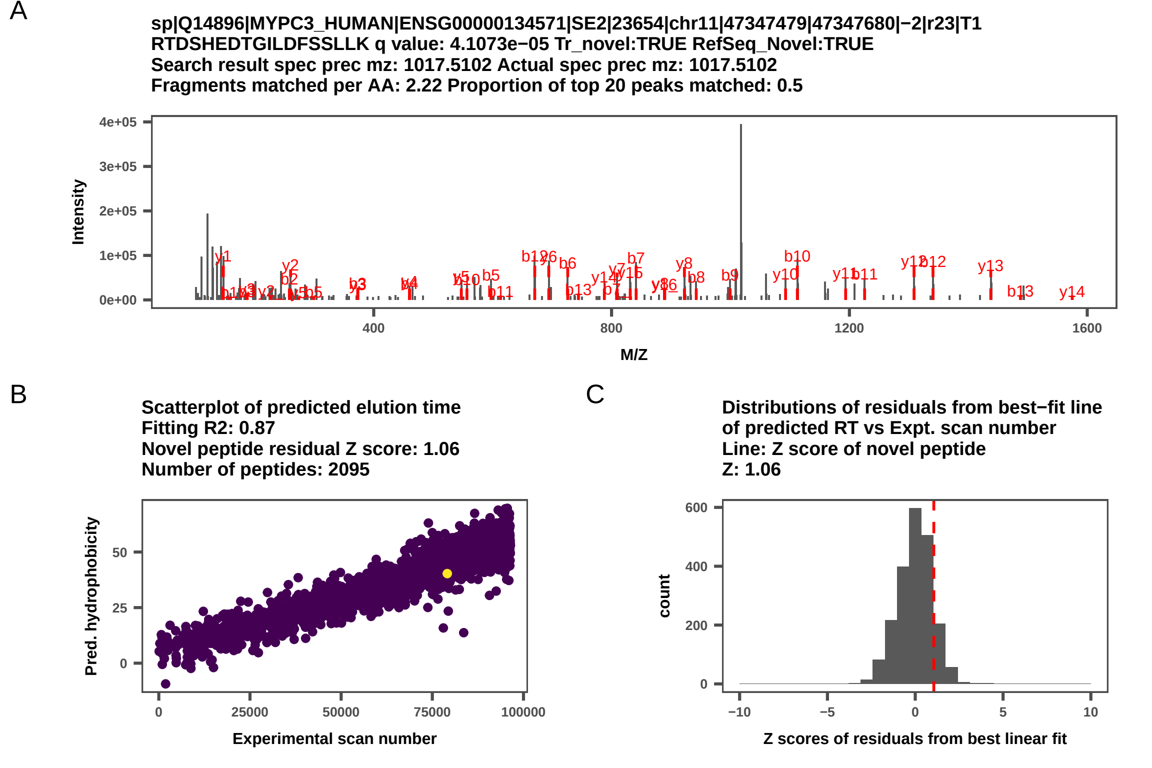
A
sp|Q14896|MYPC3_HUMAN|ENSG00000134571|SE2|23654|chr11|47347479|47347680|−2|r23|T1
RTDSHEDTGILDFSSLLK q value: 4.1073e−05 Tr_novel:TRUE RefSeq_Novel:TRUE
Search result spec prec mz: 1017.5102 Actual spec prec mz: 1017.5102
Fragments matched per AA: 2.22 Proportion of top 20 peaks matched: 0.5
Intensity
4e+05
3e+05
2e+05
1e+05
0e+00
M/Z
400
800
1200
1600
B
Scatterplot of predicted elution time
Fitting R2: 0.87
Novel peptide residual Z score: 1.06
Number of peptides: 2095
Pred. hydrophobicity
50
25
0
0
25000
50000
75000
100000
Experimental scan number
C
Distributions of residuals from best−fit line
of predicted RT vs Expt. scan number
Line: Z score of novel peptide
Z: 1.06
count
600
400
200
0
−10
−5
0
5
10
Z scores of residuals from best linear fit
